# Perioperative Hereditary Angioedema Triggered by Laryngeal Mask Airway: A Case Report and Anesthetic Implications

**DOI:** 10.7759/cureus.99886

**Published:** 2025-12-22

**Authors:** Riccardo Pulitanò, Francesca Romana Misiti, Letizia Isidori, Marco Giudice, Francesca La Verde

**Affiliations:** 1 Unit of Anesthesia and Perioperative Medicine, Azienda Ospedaliera San Giovanni Addolorata, Rome, ITA; 2 Unit of Intensive Care, Azienda Ospedaliera San Giovanni Addolorata, Rome, ITA

**Keywords:** airway management, anesthesia, hereditary angioedema, icatibant, perioperative management, supraglottic airway

## Abstract

Hereditary angioedema (HAE) is a rare condition characterized by episodic subcutaneous or mucosal edema, which may pose significant challenges in the perioperative setting. We report the management of a 79‑year-old male patient undergoing transurethral resection of a bladder tumor under general anesthesia with a supraglottic airway device. Postoperatively, the patient developed acute facial edema requiring prompt recognition and disease‑specific intervention. Rapid resolution was achieved, and the patient had an uneventful recovery. This case underscores the importance of perioperative awareness, early recognition, and careful planning to prevent airway complications in patients with HAE.

## Introduction

Hereditary angioedema (HAE) is a rare but potentially life‑threatening disorder, most often caused by deficiency or dysfunction of C1 esterase inhibitor (C1‑INH), leading to unregulated activation of the kallikrein-kinin system and excessive production of bradykinin. This results in increased vascular permeability and episodic, self‑limiting subcutaneous or submucosal edema [1]. When swelling involves the oropharynx or larynx, airway obstruction can occur rapidly, presenting a critical challenge for perioperative management, especially under anesthesia. Unlike histamine‑mediated allergic reactions, HAE attacks do not respond reliably to antihistamines, corticosteroids, or epinephrine, underscoring the need for disease‑specific therapy [2,3].

In the perioperative context, a variety of triggers, including surgical trauma, mucosal irritation, emotional stress, and airway manipulation, may precipitate an acute attack [2,4,5]. Given the rarity of HAE and the fact that a significant proportion of patients may carry de novo mutations without family history, preoperative diagnosis is often lacking [4]. This may lead to unexpected airway complications, especially when supraglottic devices or endotracheal intubation are used [5,6]. Here, we describe a case of postoperative perioral angioedema in a patient with previously unrecognized HAE, triggered by laryngeal mask airway (LMA) insertion, and discuss anesthetic considerations in light of current evidence [5,7].

## Case presentation

A 79‑year-old man with a history of hypertension on angiotensin-converting enzyme inhibitor therapy (ACE INH) and type II diabetes mellitus (American Society of Anesthesiologists physical status - ASA - 2) was scheduled for transurethral resection of bladder (TURB). Preoperative assessment did not reveal any personal or family history of angioedema, and there were no previous anesthetic complications. Given technical difficulties for spinal anesthesia, after obtaining informed consent, the team opted for general anesthesia (GA). Induction was performed with propofol 130 mg and remifentanil via target‑controlled infusion (TCI, effect‑site 1.5 ng/mL), and a size 4 I-gel LMA was placed without apparent difficulty. Anesthesia was maintained with desflurane and remifentanil (TCI 2-3 ng/mL). The procedure lasted about 60 minutes, with stable intraoperative hemodynamics and no signs of airway trauma.

**Figure 1:**
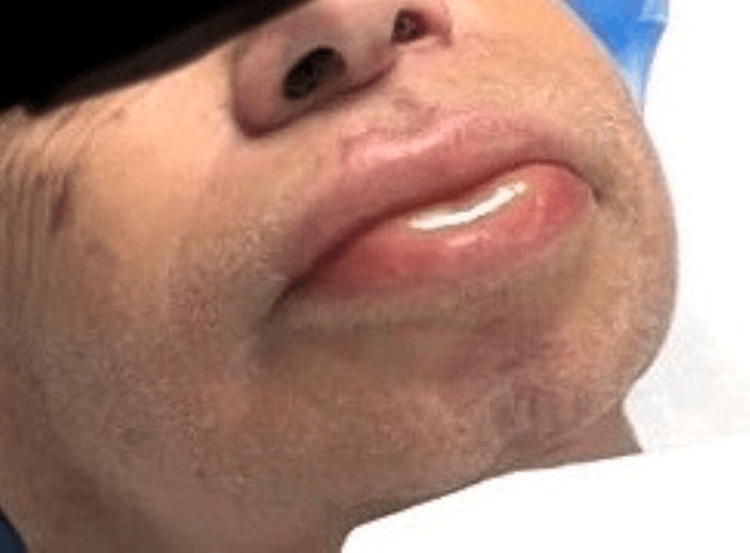
Perioral edema Marked perioral edema involving both the upper and lower lips.

Fifteen minutes after transfer to the post‑anesthesia care unit (PACU), the patient developed perioral edema involving both upper and lower lips (Figure 1).

Because of the acute onset, an allergic reaction was initially suspected and chlorphenamine 10 mg IV plus dexamethasone 8 mg IV were administered. There was no respiratory compromise, stridor, or hemodynamic instability. Subsequently, the patient revealed a history of HAE. Given this, he was transferred to the intensive care unit (ICU) for close observation and subcutaneous icatibant 30 mg was administered. Over the next two hours, the perioral swelling regressed progressively. No airway intervention was required. The patient was transferred to the hospital ward the following day and was eventually discharged without further complications [8].

To confirm the diagnostic hypothesis, which had already been supported by the patient's pre-existing clinical documentation, laboratory testing of C1-Inhibitor (C1-) levels was conducted in the subsequent days, with results showing levels below the normal range.

## Discussion

HAE results from insufficient functional C1‑INH, which normally regulates activation of the complement and contact (kallikrein-kinin) systems. In its absence or dysfunction, excessive bradykinin is generated, promoting vasodilation and increased vascular permeability; bradykinin B2 receptor activation on endothelial cells underlies the localized edema [2,3]. This mechanism explains why antihistamines or corticosteroids, effective in histamine‑mediated allergic reactions, are generally ineffective in HAE‑related swelling [3].

Surgical stress, tissue manipulation, airway instrumentation (even via supraglottic devices like LMA), mucosal irritation, and emotional stress may all act as triggers for an HAE attack [2,4,5]. The present case demonstrates how even “minor” airway manipulation, i.e., insertion of an LMA, may be sufficient to precipitate perioral edema [5,7].

Perioperative care of HAE patients requires familiarity with HAE‑specific risks and therapies. Retrospective and prospective studies emphasize the need for awareness and readiness for airway emergencies [4,5]. Guidelines highlight the importance of both on‑demand and prophylactic therapy in the perioperative period, depending on risk stratification and planned airway manipulation [5-7].

Prophylactic administration of C1‑INH concentrate prior to elective procedures has been associated with reduction in perioperative angioedema attacks [5,7]. Fresh frozen plasma (FFP) can also be used in emergencies if C1‑INH is unavailable, as it contains functional C1‑INH, though it is not first‑line therapy [6,7]. These experiences suggest that regional anesthesia when feasible, combined with prophylaxis, may reduce the risk of perioperative HAE exacerbations [4,5].

In our patient, no prophylaxis was administered because HAE was unknown; thus, no preventive measures could be taken and the airway manipulation via an LMA likely acted as a trigger. This underscores the importance of a detailed preoperative history probing for any prior unexplained episodes of facial, tongue or airway swelling, even if not formally diagnosed, and family history [4,5,7].

In the event of an acute HAE attack, prompt administration of disease‑specific therapy is critical. Available options include plasma‑derived or recombinant C1‑INH concentrate, and bradykinin B2‑receptor antagonists, such as icatibant [2,3,8]. In the present case, subcutaneous icatibant resulted in rapid resolution of perioral edema within two hours, with no airway compromise [8].

While some literature reports use of icatibant also for non‑hereditary or drug‑induced angioedema (e.g., ACEi-related) with benefit, data in the perioperative context are more scarce. Hence, it is recommended that anesthesiologists ensure availability of on-demand therapy (C1‑INH concentrate or icatibant), FFP as backup, and that the “difficult airway” cart remain immediately accessible throughout the perioperative period [4,5,7].

Based on our experience and the literature, we propose the following pragmatic recommendations for anesthetic management of (known or suspected) HAE patients: on the *preoperative phase,* conduct a targeted history including previous episodes of unexplained swelling, family history, known triggers and consider preoperative prophylaxis with C1‑INH concentrate or appropriate long‑term prophylaxis in relation to the patient profile and type of procedure [4,5,7]; for *anesthetic planning, *prefer regional or local anesthesia when feasible and, If GA is necessary, minimize airway manipulation [5,7]; for *airway preparedness, *ensure videolaryngoscope, fiber‑optic scope and surgical airway kit [5,7]; for *therapy readiness, *ensure on‑demand therapy (C1‑INH concentrate, icatibant) and, if necessary, FFP as backup [2,3,8]; for *postoperative care, ensure* monitoring in ICU for at least 24-48 hours, with low threshold for early intervention [4,5,7].

## Conclusions

HAE remains a potentially under‑recognized but dangerous risk in the perioperative period. Even seemingly benign airway manipulation, such as insertion of an LMA, may trigger a bradykinin‑mediated angioedema attack with risk of airway compromise. A high index of suspicion, careful preoperative assessment, prophylactic measures when indicated, gentle airway management, ready availability of disease‑specific therapy, and vigilant postoperative monitoring are essential to ensure patient safety.

## References

[1] Williams AH, Craig TJ (2015). Perioperative management for patients with hereditary angioedema. Allergy Rhinol (Providence).

[2] MacBeth LS, Volcheck GW, Sprung J, Weingarten TN (2016). Perioperative course in patients with hereditary or acquired angioedema. J Clin Anesth.

[3] Tanaka KA, Mondal S, Morita Y, Williams B, Strauss ER, Cicardi M (2020). Perioperative management of patients with hereditary angioedema with special considerations for cardiopulmonary bypass. Anesth Analg.

[4] Hosokawa R, Tsukamoto M, Nagano S, Yokoyama T (2019). Anesthetic management of a patient with hereditary angioedema for oral surgery. Anesth Prog.

[5] Bang YS, Cho J, Park C (2022). An anesthetic experience of hereditary angioedema type I patient undertook total laparoscopic hysterectomy - a case report. Anesth Pain Med (Seoul).

[6] Aygören-Pürsün E, Martinez Saguer I, Kreuz W, Klingebiel T, Schwabe D (2013). Risk of angioedema following invasive or surgical procedures in HAE type I and II--the natural history. Allergy.

[7] Robledo PG, Ortiz JC (2025). Anaesthesetic considerations in the perioperative management of patients with hereditary angioedema-FXII. Rev Esp Anestesiol Reanim (Engl Ed).

[8] Estevens TM, Serrano A, Amaro S, Ribeiro J (2020). Peri-operative management of a pregnant patient with hereditary angioedema submitted to a cesarean-section: case report (Article in Portuguese). Braz J Anesthesiol.

